# Clinico-molecular characteristics and enhanced efficacy of immune checkpoint inhibitors in *TP53*-mutated advanced biliary tract cancer

**DOI:** 10.1007/s00535-026-02439-9

**Published:** 2026-05-25

**Authors:** Taro Shibuki, Celine Hoyek, Ryne Ramaker, Madhuri Gottam, Makoto Ueno, Yasuyuki Kawamoto, Shinji Itoh, Kanae Inoue, Tadayoshi Hashimoto, Takao Fujisawa, Mitsuho Imai, Yoshiaki Nakamura, Hideaki Bando, Angelo Pirozzi, Binbin Zheng-Lin, Oluseyi Abidoye, Cody R. Eslinger, Mohamad Bassam Sonbol, Hani Babiker, Nguyen Tran, Chigusa Morizane, John H. Strickler, Takayuki Yoshino, Tanios Bekaii-Saab, Masafumi Ikeda

**Affiliations:** 1 Department for the Promotion of Drug and Diagnostic Development, Division of Drug and Diagnostic Development Promotion, Translational Research Support Office, National Cancer Center Hospital East, 6-5-1, Kashiwanoha, Kashiwa, Chiba 277-8577 Japan; 2 Department of Hepatobiliary and Pancreatic Oncology, National Cancer Center Hospital East, Kashiwa, Japan; 3 Department of Hematology and Oncology, Mayo Clinic, Arizona, AZ USA; 4 Duke Cancer Institute, Durham, NC USA; 5 Department of Gastroenterology, Kanagawa Cancer Center, Yokohama, Japan; 6 Division of Cancer Center, Hokkaido University Hospital, Sapporo, Japan; 7 Department of Hepatobiliary and Pancreatic Oncology, National Cancer Center Hospital, Tokyo, Japan; 8 Department of Surgery and Science, Graduate School of Medical Sciences, Kyushu University, Fukuoka, Japan; 9 Department of Gastroenterology and Gastrointestinal Oncology, National Cancer Center Hospital East, Kashiwa, Japan; 10 Department of Head and Neck Medical Oncology, National Cancer Center Hospital East, Kashiwa, Japan; 11 Earle A. Chiles Research Institute, Providence Cancer Institute, Portland, OR USA; 12 Department of Hematology and Oncology, Mayo Clinic, Jacksonville, FL USA; 13 Department of Oncology, Mayo Clinic, Rochester, MN USA

**Keywords:** Advanced biliary tract cancer, *TP53*, Prognosis, Systemic therapy, Immune checkpoint inhibitor

## Abstract

**Background:**

*TP53* mutations are among the most frequent alterations in biliary tract cancer (BTC), but their prognostic relevance and predictive value for immune checkpoint inhibitors (ICI) remain unclear. We assessed clinico-molecular features, prognosis, and ICI efficacy by *TP53* status in advanced BTC.

**Methods:**

Patients with advanced BTC from Japan and the United States who underwent tissue- or plasma-based next-generation sequencing were retrospectively analyzed. Tissue-based sequencing was used for the primary analysis, and plasma ctDNA sequencing was evaluated as an exploratory cohort. Outcomes were compared between *TP53* wild-type (WT) and *TP53*-mutated groups. Treatment–*TP53* interactions for non-ICI versus ICI-containing regimens were assessed using multivariable Cox models. Whole-transcriptome sequencing data were analyzed using TIDE and Hallmark gene set enrichment analysis.

**Results:**

Among 336 patients in the tissue-based cohort, 177 (52.7%) had *TP53*-mutated tumors. *SMAD4, ERBB2, PTEN* and *CCNE1* co-alterations were enriched in *TP53*-mutated tumors, whereas *IDH1, BAP1*, and *FGFR2* fusions were more frequent in WT tumors. *TP53* mutations were independently associated with shorter PFS, whereas OS showed a similar but nonsignificant trend (PFS: HR 1.32, *P* = 0.04; OS: HR 1.32, *P* = 0.09). *TP53* mutations were associated with shorter PFS in the non-ICI cohort but numerically longer PFS in the ICI cohort, with a significant treatment–*TP53* interaction for PFS (*P* < 0.01). Similar trends were observed in the plasma cohort. Transcriptomic analyses (*n* = 59; all MSS) showed lower TIDE scores in *TP53*-mutated tumors.

**Conclusion:**

*TP53* mutations were associated with poor prognosis and may predict greater benefit from ICI, supporting biomarker-driven stratification.

**Supplementary Information:**

The online version contains supplementary material available at https://doi.org/10.1007/s00535-026-02439-9.

## Introduction

Biliary tract cancer (BTC) comprises a diverse group of malignancies, including intrahepatic and extrahepatic cholangiocarcinoma (ICC, ECC), gallbladder carcinoma (GBC) and ampullary carcinoma (AC). The incidence of BTC varies widely by subtype and geographic region, with cholangiocarcinoma showing a rising trend in high-income countries (0.35–2/100,000 annually) and an endemic prevalence in Southeast Asia, where incidence rates are up to 40 times higher [1]. While surgical resection remains the only curative treatment, most patients present with advanced disease, precluding surgery due to distant metastases or locoregional factors such as vascular invasion [2]. Even after curative-intent resection, recurrence is common [3]. Consequently, systemic therapies are the cornerstone of BTC treatment.

Recent advances in systemic therapy have improved outcomes for patients with advanced BTC. The combination of gemcitabine and cisplatin (GC) with immune checkpoint inhibitors (ICI) has demonstrated superior disease control compared to conventional chemotherapy [4, 5]. However, clinical benefits remain modest, with a hazard ratio (HR) of approximately 0.8, and only a subset of patients derive significant benefit [4, 5]. The underlying determinants of treatment response remain poorly understood, emphasizing the need for predictive biomarkers to guide therapeutic strategies.

Among potential biomarkers, PD-L1 shows limited predictive value in BTC. In the KEYNOTE-158 study, while 58% of patients had PD-L1 ≥ 1%, only 6% exhibited a clinical response. The objective response rate (ORR) was 6.6% in PD-L1-positive and 2.9% in PD-L1-negative patients, with median progression-free survival (PFS) of 1.9 and 2.1 months, respectively [6]. DNA damage repair (DDR) gene mutations are candidate biomarkers for ICI efficacy. Approximately 25% of BTC may harbor DDR deficiencies [7], yet evidence for ICI efficacy remains limited. In a study of 86 patients with solid tumors, only 4 patient with BTC received pembrolizumab. Although the ORR was 25%, the small sample size makes it difficult to draw firm conclusions [8]. Similarly, microsatellite instability (MSI) and deficient mismatch repair (dMMR) have been explored as predictive markers. The KEYNOTE-158 study assessed pembrolizumab in advanced MSI-High (MSI-H)/dMMR tumors, including 22 BTC patients. The ORR was 40.9%, with 2 complete and 7 partial responses. Median PFS was 4.2 months, and median overall survival (OS) reached 24.3 months [9]. Despite MSI being a well-established biomarker, its prevalence in BTC is low (< 2%), and response to ICI remains inconsistent [10].

*TP53* is among the most frequently mutated genes across malignancies and is central to genomic stability. Its mutations carry prognostic implications in many cancers, including BTC, and have been associated with response to ICI in NSCLC [11]. Although *TP53* mutations are common in BTC, their prognostic and predictive value for ICI therapy remains unclear. To address this knowledge gap, we leveraged large-scale clinical and genomic datasets to investigate the prognostic impact of *TP53* mutations in BTC and to explore their association with outcomes following ICI treatment.

## Methods

### Patients and study design

This retrospective observational study was conducted within the ONCOJACK framework, an international collaborative network integrating clinical and molecular data from patients with malignant tumors. Patients with unresectable locally advanced or metastatic BTC, including ICC, ECC, GBC, and AC, were included. Data were retrospectively collected from Japan and the U.S. In Japan, patients were identified from the SCRUM-Japan GOZILA and MONSTAR-SCREEN studies or were treated at the NCCHE. In the United States, patients were selected through clinically annotated medical records from the Mayo Clinic Comprehensive Cancer Center (Arizona, Minnesota, and Florida) and Duke Cancer Institute (North Carolina). The inclusion criteria were as follows: (1) histologically or cytologically confirmed advanced BTC; (2) age ≥ 18 years; (3) receipt of any palliative systemic therapy; and (4) completion of comprehensive genomic profiling.

Genomic alterations were detected using tissue-based next-generation sequencing (NGS) platforms, including FoundationOne CDx (Foundation Medicine), Caris MI Profile (Caris Life Sciences), and the OncoGuide NCC Oncopanel System (Sysmex Corporation). Plasma ctDNA-based NGS platforms, such as Guardant360 CDx (Guardant Health) and FoundationOne Liquid CDx (Foundation Medicine), were also utilized. *TP53* mutation status was determined based on *TP53* alterations reported by each clinically validated platform. Variants classified as variants of uncertain significance were excluded from the analysis. All pathogenic or likely pathogenic *TP53* alterations were included if they met the respective platform-specific reporting criteria. For the primary analysis, *TP53* status was determined using tissue-based NGS results only. Patients who underwent plasma ctDNA-based NGS alone were not included in the primary analysis and were evaluated separately in the exploratory analysis. In liquid biopsy, genetic alterations with a variant allele frequency (VAF) greater than 1.0% were included. Results were reported according to the clinical thresholds defined by each platform.

The timing of comprehensive genomic profiling varied across cohorts. In the GOZILA study, plasma-based NGS was performed after disease progression on systemic therapy. In MONSTAR-SCREEN and MONSTAR-SCREEN-2, comprehensive genomic profiling was generally performed before first-line systemic therapy. In the National Cancer Center Hospital East cohort, profiling was performed in routine clinical practice under the Japanese insurance system, typically after failure of standard therapy. In the Mayo Clinic and Duke Cancer Institute cohorts, profiling was performed as part of routine clinical care before or during systemic treatment, according to institutional practice and tumor specimen availability.

The study was approved by local Ethics Committee at each center, and conducted in accordance with the protocol, the Declaration of Helsinki. Study protocols were approved by the institutional review boards (IRB) of participating institutions and registered in the University Hospital Medical Information Network (UMIN) Clinical Trials Registry (protocol IDs: GOZILA: UMIN000029315, MONSTAR-SCREEN: UMIN000036749, MONSTAR-SCREEN-2: UMIN000043899). Written informed consent was obtained from patients enrolled in the SCRUM-MONSTAR-SCREEN project. For retrospectively collected patient data, informed consent was obtained using an opt-out approach in accordance with institutional policies. This international integrated analysis study was approved by the IRB of the National Cancer Center Hospital East (NCCHE) (UMIN000049334).

### Genomic co-alteration analysis

Genomic co-alteration analysis was performed in the tissue-based cohort. The frequencies of recurrent genomic alterations reported by each platform were compared between *TP53* mutation and WT groups. Differences in alteration frequencies between the groups were assessed using Fisher’s exact test.

### Analysis of the molecular pathways and response to immunotherapy

To explore the molecular and immunologic features associated with *TP53* mutations in BTC, we analyzed whole-transcriptome sequencing (WTS) data from the MONSTAR-SCREEN-2 study. The Tumor Immune Dysfunction and Exclusion (TIDE) algorithm (http://tide.dfci.harvard.edu) [12] was applied to predict immunotherapy responsiveness. Higher TIDE scores reflect a greater likelihood of immune evasion and poorer ICI response, whereas lower scores indicate improved responsiveness. In parallel, gene set enrichment analysis (GSEA) was performed using Hallmark gene sets from the Molecular Signatures Database (MSigDB; h.all.v2024.1.Hs.symbols.gmt), with enrichment defined as Family-Wise Error Rate *P* value < 0.05 [13].

### Assessments

**Fig. 1:**
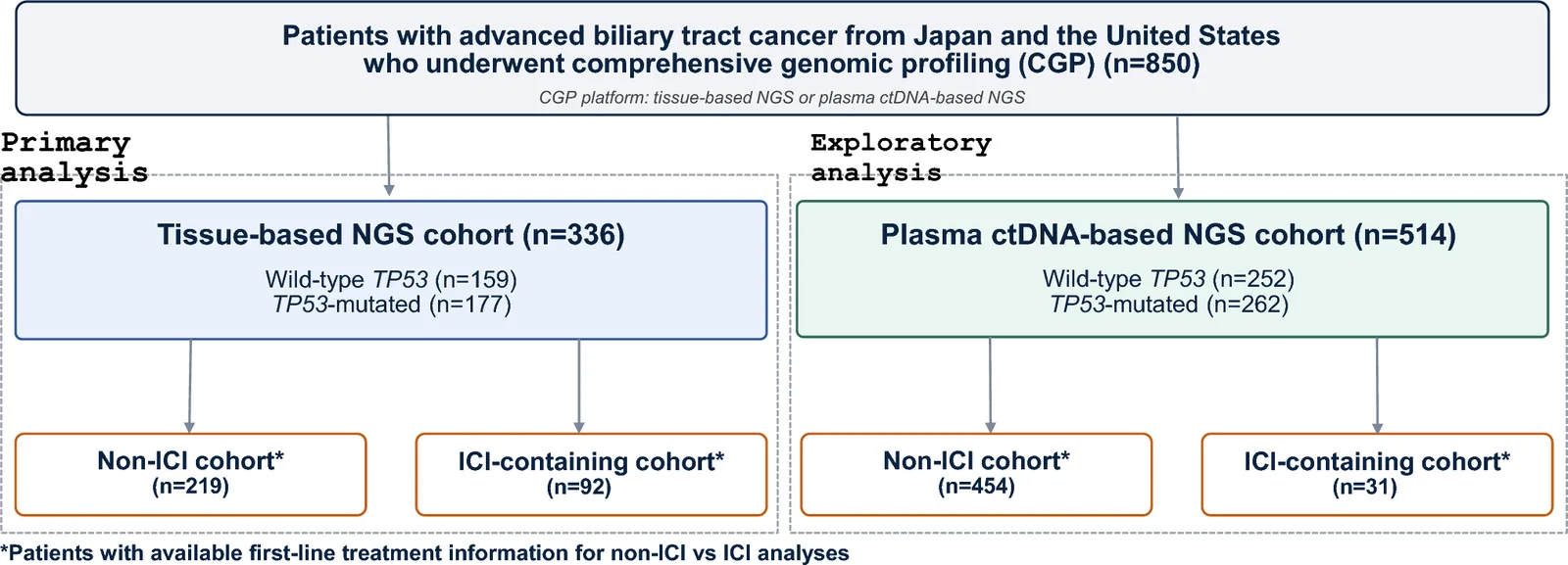
Flow diagram of the study patients. BTC, biliary tract cancer; ICI, immune checkpoint inhibitor

All efficacy analyses were restricted to patients receiving first-line systemic therapy. Two cohorts were used (Fig. 1): a prognostic cohort of advanced BTC to compare outcomes by *TP53* status (mutated vs wild-type [WT]), and a first-line comparative cohort to compare non-ICI and ICI regimens. Tumor response was evaluated radiologically using CT/MRI in line with RECIST version 1.1 criteria [14]. An objective response was defined as a complete or partial response, and disease control was defined as a complete response, partial response, or stable disease as the best response. The OS was measured from the date of initiation of first-line treatment to the date of death from any cause or date of the last follow-up, or censored at the final follow-up examination. The PFS was defined as the period from the date of initiation of first-line treatment to the date of documentation of disease progression or death from any cause, or censored at the final follow-up examination.

### Statistical analysis

Categorical variables were compared with Fisher’s exact test and continuous variables with Mann–Whitney U or Kruskal–Wallis tests. OS and PFS were estimated by the Kaplan–Meier method and compared using the log-rank test. Prognostic factors were assessed by multivariable Cox proportional hazards analysis. The interaction effects between the treatment regimen and key covariates were tested within the multivariable Cox models by including multiplicative interaction terms, and the corresponding *P* values were reported as *P* for interaction. Statistical significance was set at *P* < 0.05, and analyses were conducted using R (version 4.4.0, R Foundation, Vienna, Austria).

## Results

### Patient characteristics

In the primary tissue-based cohort, a total of 336 patients were included (WT: 159, *TP53* mutation: 177; Table 1). Patients in the *TP53* mutation group were more likely to have ECC (29.9% vs. 13.2%) or GBC (31.6% vs. 20.1%), whereas ICC was more frequent in the WT group (59.7% vs. 30.5%; *P* < 0.01). Liver metastasis was also more common in the *TP53* mutation group (50.8% vs. 39.0%; *P* = 0.04). No significant differences were observed between the two groups in age, sex, tumor status, MSI-High frequency, other metastatic sites, geographic location, or distribution of first-line treatment regimens.

**Table 1:** Baseline characteristics

	WT (*n* = 159)	*TP53-*mutated (*n* = 177)	*P* value
Age (years), *n* (%)			
Median [range]	67 [28, 83]	69 [26, 84]	0.09
< 70	95 (60.1)	94 (53.1)	0.24
≥ 70	64 (39.9)	83 (46.9)	
Sex, *n* (%)			
Male	82 (51.6)	93 (52.5)	0.91
Female	77 (48.4)	84 (47.5)	
Primary tumor site, *n* (%)			
Extrahepatic bile duct	21 (13.2)	53 (29.9)	< 0.01
Gallbladder	32 (20.1)	56 (31.6)	
Intrahepatic bile duct	95 (59.7)	54 (30.5)	
Ampulla of Vater	11 (6.9)	14 (7.9)	
Tumor status, *n* (%)			
Recurrence	51 (32.1)	61 (34.5)	0.60
Locally advanced	13 (8.2)	19 (10.7)	
Metastatic	95 (59.7)	97 (54.8)	
MSI-high detected, n (%)	3 (2.0)	4 (2.3)	1
Site of metastasis, n (%)			
Liver	62 (39.0)	90 (50.8)	0.04
Lymph node	57 (35.8)	62 (35.0)	0.91
Lung	28 (17.6)	26 (14.7)	0.55
Peritoneum	22 (13.8)	17 (9.6)	0.24
Others	7 (4.4)	9 (5.1)	0.80
Site, n (%)			
Japan	135 (84.9)	153 (86.4)	0.81
US	24 (15.1)	24 (13.6)	
1 st line regimen			
Triplet	86 (54.1)	106 (59.9)	0.53
Gem + Cis + S-1	44 (27.7)	58 (32.8)	
Gem + Cis + ICI	42 (26.4)	48 (27.1)	
Doublet	55 (34.6)	58 (32.8)	
Gem + Cis	52 (32.7)	53 (29.9)	
Gem + S-1	3 (1.9)	5 (2.8)	
Singlet	3 (1.9)	3 (1.7)	
S-1	1 (0.6)	0 (0)	
Gem	2 (1.3)	1 (0.6)	
ICI	0 (0)	2 (1.1)	
Unknown	15 (9.4)	10 (5.6)	

WT, wild-type; MSI, microsatellite status instability; NGS, next-generation sequencing; Gem, gemcitabine; Cis, cisplatin; ICI, immune checkpoint inhibitor

In the exploratory plasma ctDNA-based cohort, 514 patients were included: 252 in the WT group and 262 in the *TP53* mutation group (Supplementary Table S1). Patients in the *TP53* mutation group were older and more likely to have GBC and liver metastasis, whereas ICC was more frequent in the WT group.

### Genomic co-alterations with or without *TP53* mutations and distribution of *TP53* variants in tissue-based NGS

**Fig. 2:**
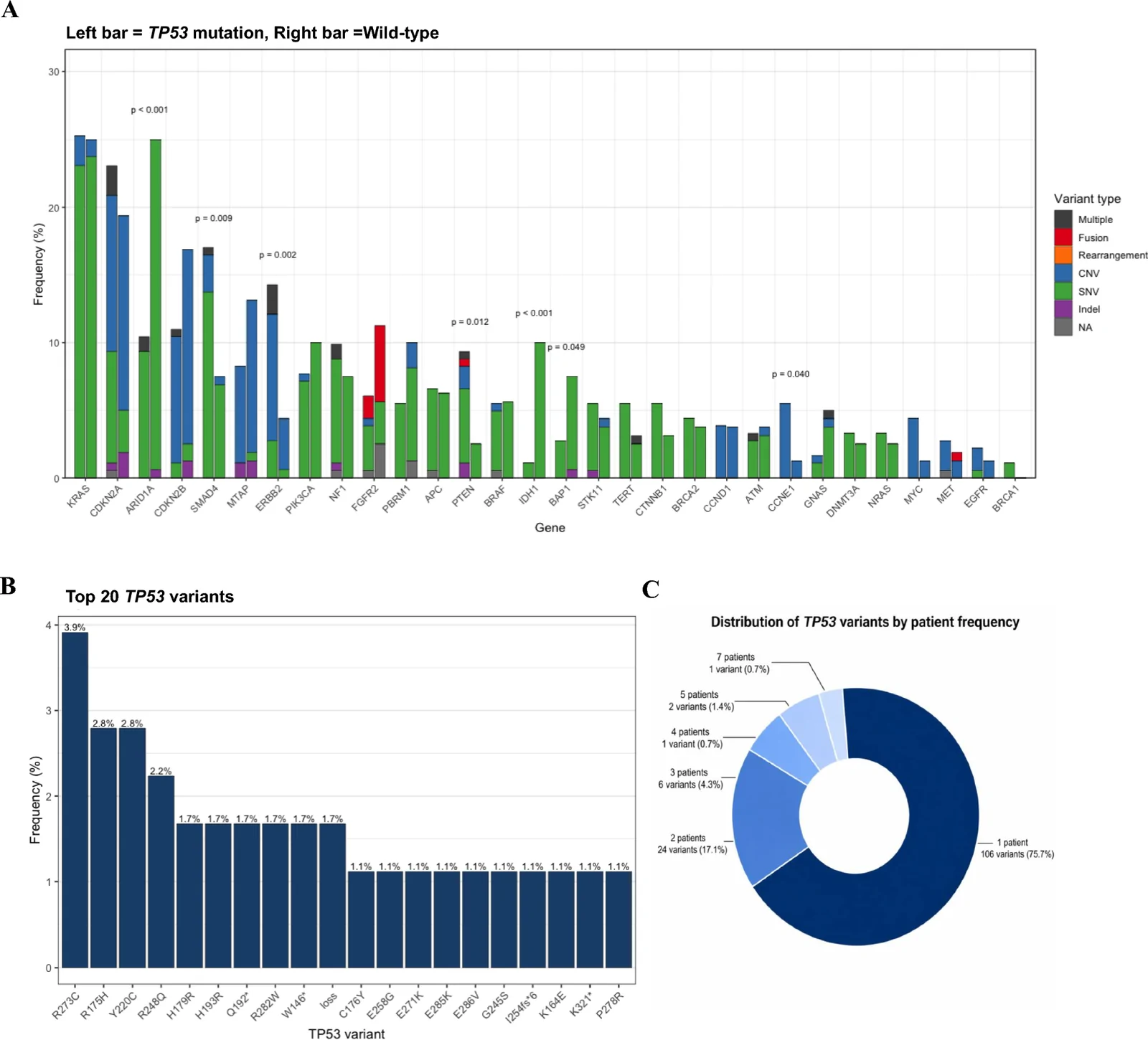
A Comparison of the genomic alterations in patients with and without *TP53* mutations. B Distribution of the top 20 most frequently encountered *TP53* variants among patients with *TP53* mutations. C Distribution of *TP53* variants by patient frequency

Genomic co-alterations differed significantly by *TP53* status (Fig. 2A). Compared with WT, the *TP53* mutation group more often harbored *SMAD4*, ERBB2, *PTEN*, and *CCNE1* alterations (all *P* < 0.05), whereas *IDH1* and *BAP1* mutations, and *FGFR2* fusion were enriched in the WT group (all *P* < 0.05). Among the *TP53* mutation group, the most frequent variant was R273C, followed by R175H, Y220C and R248Q (Fig. 2B). The distribution of *TP53* variants by patient frequency is shown in Fig. 2C. Most variants were infrequent and observed in only one patient (106 variants, 75.7%), whereas 24 variants (17.1%) were observed in two patients, and only a small number of variants were shared by three or more patients.

### Impact of *TP53* mutations on the treatment response and survival

**Fig. 3:**
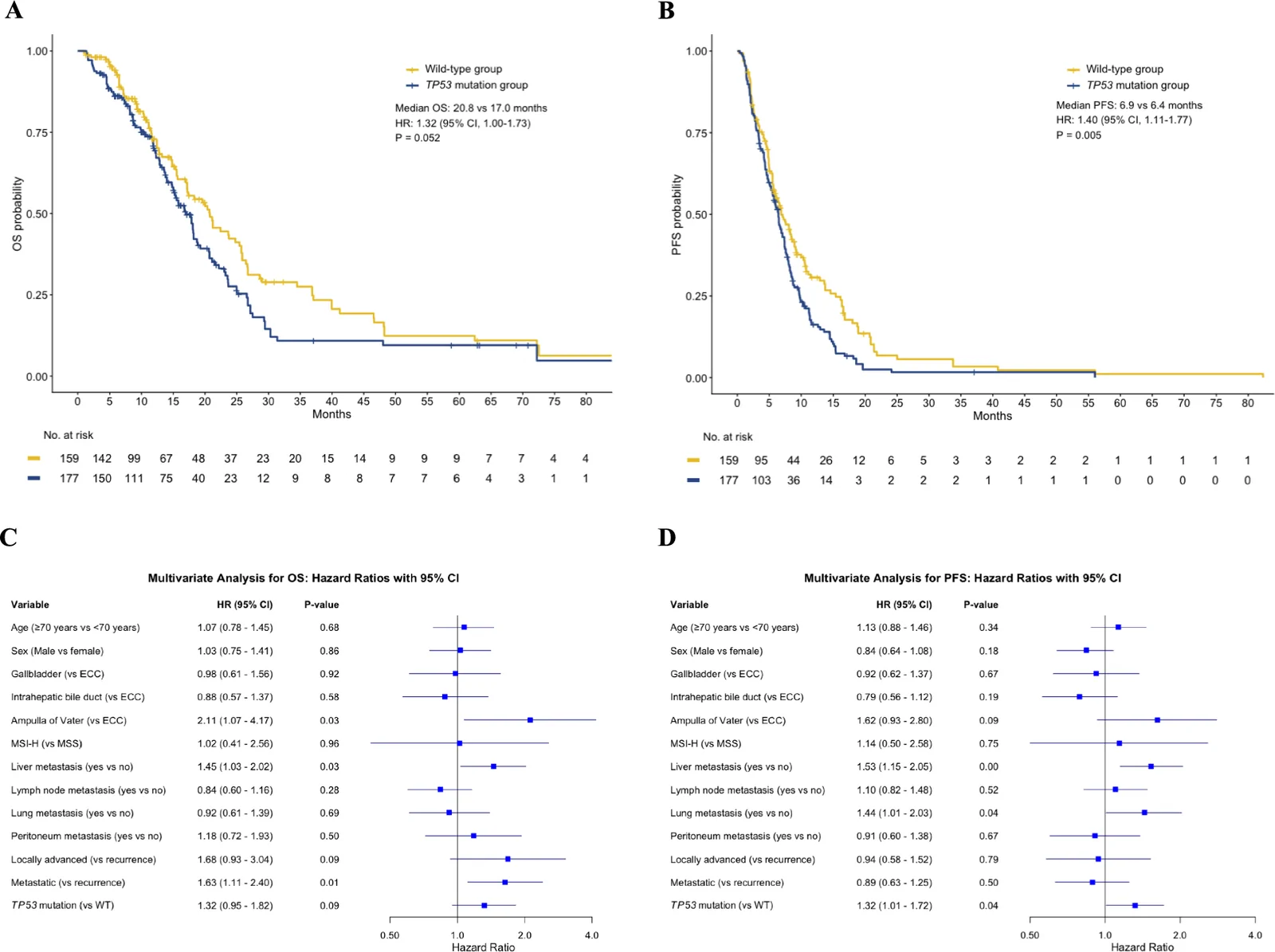
Kaplan–Meier curves for OS (**A**) and PFS (**B**) according to the tissue-based *TP53* mutation status in patients treated with first-line systemic therapy, and multivariable analyses conducted using Cox proportional hazards models to identify factors predictive of the OS (**C**) and PFS (**D**) in patients with advanced biliary tract cancer. OS, overall survival; PFS, progression-free survival; ECC, extrahepatic cholangiocarcinoma; MSI-H, microsatellite instability-high; MSS, microsatellite-stable; WT, wild-type

Treatment response and survival following first-line systemic therapy were evaluated in the tissue-based cohort. First-line regimens were similar by *TP53* status (overall *P* = 0.53). Most patients received GC (WT vs *TP53* mutation, 34.6% vs 38.2%), followed by GC + S-1 (27.7% vs. 32.8%) and GC + ICI (26.4% vs. 27.1%) (Table 1). Treatment responses were similar by *TP53* status: WT vs *TP53* mutation, ORR 27.7% vs 26.4% (*P* = 0.89) and DCR 76.3% vs 73.0% (*P* = 0.57). However, median OS was shorter in the *TP53* mutation group, although the difference did not reach statistical significance (20.8 vs. 17.0 months, HR 1.32, 95% confidence interval [CI] 1.00–1.73; *P* = 0.05, Fig. 3A). Similarly, median PFS was significantly worse in the *TP53* mutation group (6.9 vs. 5.4 months, HR 1.40, 95% CI 1.11–1.77; *P* < 0.01, Fig. 3B). In multivariable analysis, *TP53* mutations were not independently associated with shorter OS (HR 1.32; *P* = 0.09, Fig. 3C), but were independently associated with shorter PFS (HR 1.32; *P* = 0.04, Fig. 3D). In a sensitivity analysis excluding patients with *IDH1* mutations or *FGFR2* fusions, the direction of association remained consistent after multivariable adjustment, although statistical significance was not reached (OS: adjusted HR, 1.22; *P* = 0.25; PFS: adjusted HR, 1.31; *P* = 0.06).

Similar trends were observed in the exploratory plasma ctDNA-based cohort. Treatment responses did not differ significantly by *TP53* status, with ORR of 21.1% versus 19.1% (*P* = 0.64) and DCR of 66.9% versus 63.4% (*P* = 0.45) in the WT and the *TP53* mutation groups, respectively. Patients in the *TP53* mutation group had significantly shorter OS (16.0 vs. 21.0 months; HR 1.28; 95% CI 1.02–1.61; *P* = 0.031) and PFS (5.7 vs. 6.8 months; HR 1.24; 95% CI 1.02–1.50; *P* = 0.030) than those in the WT group (Fig. S1).

### Efficacy results for non-ICI vs. ICI-containing regimens according to *TP53* status

**Fig. 4:**
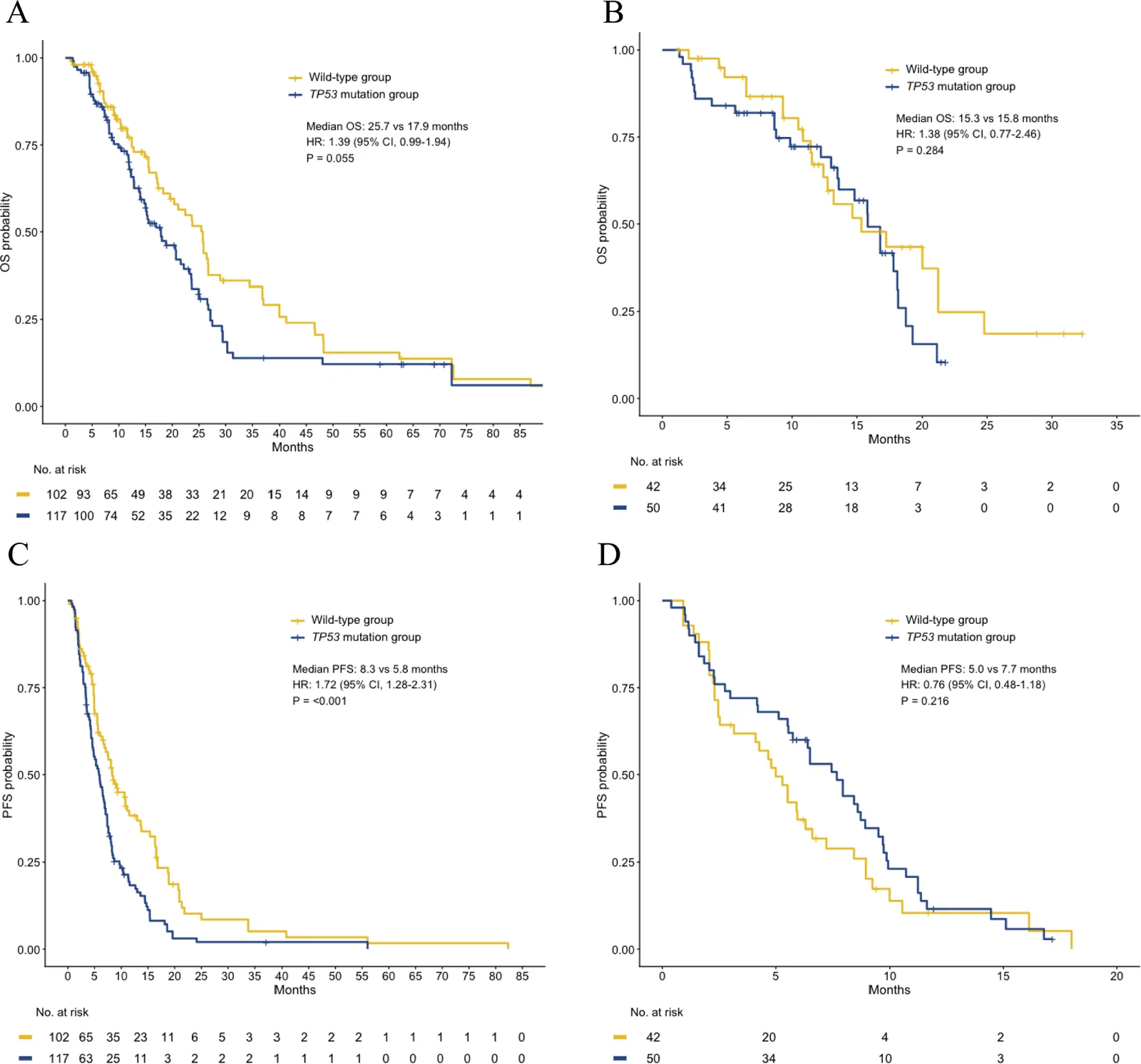
Kaplan–Meier survival curves for OS and PFS according to tissue-based *TP53* mutation status in patients with advanced biliary tract cancer stratified by first-line treatment. In regard to the OS, among the patients who received non-ICI regimens, the *TP53* mutation group showed numerically shorter survival than the WT group (median OS: 17.9 vs. 25.7 months, *P* = 0.055; (**A**), whereas no significant difference between these two patient groups was observed among the patients who received ICI-containing regimens (median OS: 15.8 vs. 15.3 months, *P* = 0.284; B). In regard to the PFS, among the patients who received non-ICI regimens, the *TP53* mutation group showed significantly shorter PFS than the WT group (median PFS: 5.8 vs. 8.3 months, *P* < 0.001; (**C**), whereas among the patients treated with ICI-containing regimens, the *TP53* mutation group showed no significant difference in PFS, although a numerically longer PFS was observed (median PFS: 7.7 vs. 5.0 months, *P* = 0.216; (**D**). OS, overall survival; PFS, progression-free survival; WT, wild-type; HR, hazard ratio; CI, confidence interval

**Fig. 5:**
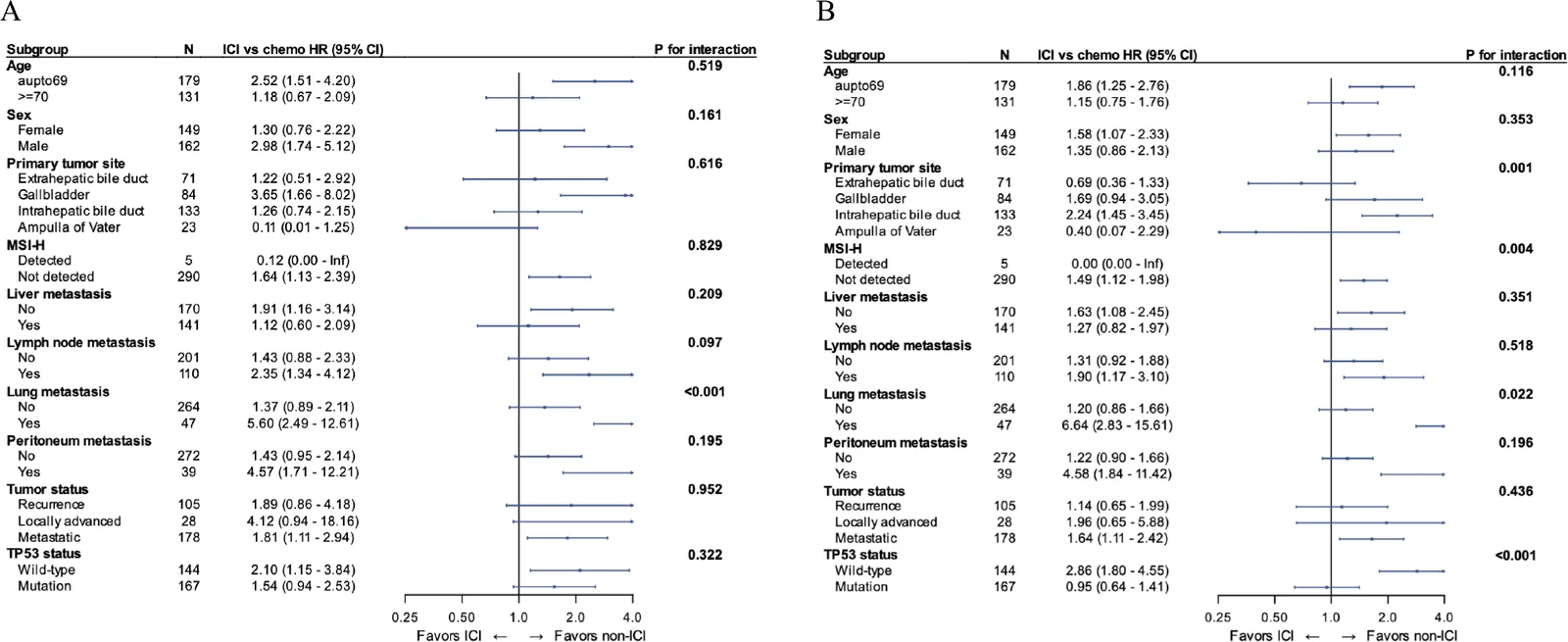
Forest plot of the treatment effect on the PFS (A) and OS (B) in subgroup analyses in the tissue-based cohort. HR, hazard ratio; CI, confidence interval; MSI-H, microsatellite instability-high; MSS, microsatellite-stable; ICI, immune checkpoint inhibitor

Baseline characteristics of patients treated with non-ICI and ICI-containing regimens are shown in Supplementary Table S2. The ICI cohort included a higher proportion of female patients, and ICC, while *TP53* status was balanced between cohorts. Supplementary Figure 2 presents ORR and DCR by *TP53* status in the tissue-based cohort among patients receiving non-ICI (Fig. S2A) or ICI regimens (Fig. S2B). In the non-ICI group, ORR (27.5% vs. 23.1%) and DCR (80.4% vs. 76.9%) were comparable between the WT and the *TP53* mutation groups, showing no significant differences. In contrast, in the ICI group, the *TP53* mutation group showed a trend toward a higher ORR (21.4% vs. 32.0%, *P* = 0.35) and significantly higher DCR (47.6% vs. 70.0%, *P* = 0.03) than the WT group. Figure 4 illustrates the impact of *TP53* status on survival in the tissue-based cohort. In the non-ICI group (Fig. 4A, C), the *TP53* mutation group had numerically shorter OS and significantly shorter PFS than the WT group. Conversely, in the ICI group (Fig. 4B, D), the *TP53* mutation group showed a trend toward longer PFS, though not statistically significant, while OS remained similar between groups. Subgroup analyses demonstrated a significant interaction between treatment regimen and *TP53* status for PFS (*P* for interaction < 0.01; Fig. 5B). MSI status was also associated with differential treatment effects for PFS (*P* for interaction < 0.01). In addition, when restricted to the microsatellite-stable (MSS) population, the treatment–*TP53* interaction remained significant for PFS (HR 0.46; 95% CI 0.23–0.91; *P* for interaction < 0.001).

Findings in the plasma ctDNA-based cohort were directionally consistent with those of the tissue-based cohort. Detailed efficacy outcomes according to *TP53* status are summarized in Supplementary Table S3. Patients in the *TP53* mutation group showed poorer outcomes in the non-ICI cohort, whereas this disadvantage was not observed in the ICI group. Although ORR and DCR differences did not reach statistical significance, the same general trend was observed (Fig. S3), along with similar patterns in survival and subgroup analyses (Figs. S4 and S5).

To further evaluate whether differences in post-progression management may have influenced OS, we assessed subsequent systemic therapy after progression according to *TP53* status among patients with available post-progression treatment information. The proportion of patients receiving subsequent systemic therapy was comparable between *TP53* mutation and WT tumors in both the ICI cohort (16/20, 80.0% vs. 11/17, 64.7%; *P* = 0.460) and the non-ICI cohort (59/91, 64.8% vs. 47/84, 56.0%; *P *= 0.279) (Additional file 10: Table S4). These findings suggest that differences in post-progression treatment are unlikely to fully account for the observed survival patterns.

### Transcriptomic analyses of *TP53*-mutated BTC

Transcriptomic profiling of the MONSTAR-SCREEN-2 WTS dataset was conducted to assess immunologic and molecular features of *TP53* mutation versus WT tumors. Among the analyzed samples (*n* = 59), no MSI-H cases were identified; thus, all analyses were conducted in the MSS context. The *TP53* mutation group exhibited significantly lower TIDE scores compared with WT counterparts (*P* = 0.01; Fig. S6A, B). Furthermore, GSEA identified significant enrichment of hallmark pathways in the *TP53* mutation group, most prominently oxidative phosphorylation (NES = 2.11, FWER *P* < 0.01), epithelial–mesenchymal transition (NES = 2.07, FWER *P* < 0.01), and hypoxia (NES = 1.74, FWER *P* < 0.01) (Fig. S6C).

## Discussion

This study evaluated the clinical significance of *TP53* mutations in patients with BTC using both tissue-based and plasma ctDNA-based analyses. In the tissue-based cohort, the *TP53* mutation group showed poorer PFS and a similar trend toward shorter OS under systemic therapy and a significant treatment–*TP53* interaction for PFS, both in the overall cohort and in the MSS subset, suggesting that the *TP53* mutation group may derive greater relative benefit from ICI. In the plasma ctDNA-based cohort, the overall direction of association was broadly consistent with the tissue-based findings. The *TP53* mutation group showed shorter OS and PFS in Kaplan–Meier analyses, although these associations were attenuated in multivariable models. In subgroup analyses, a significant treatment–TP53 interaction was observed for PFS, but not for OS. To our knowledge, this is among the largest studies to characterize the prognostic impact of *TP53* mutations in advanced BTC and the first to examine their potential predictive relevance for ICI treatment across both tissue-based and plasma ctDNA-based settings.

In the tissue-based cohort, the *TP53* mutation group had shorter OS and PFS than the WT group among patients with advanced BTC treated with systemic therapy. These findings align with previous studies across various malignancies including BTC, linking *TP53* mutations to aggressive biology and resistance to standard therapies [15–19]. In this study, we confirmed this association in a larger cohort, further reinforcing the prognostic significance of *TP53* mutations. p53 is a key tumor suppressor that regulates cell cycle arrest, apoptosis, and DNA repair. *TP53* mutations not only disrupt these functions, enabling uncontrolled cell proliferation, but also promote tumor progression and metastasis [20]. For example, mutant p53 can enhance epithelial–mesenchymal transition (EMT), facilitating invasion and dissemination [21]. Consistent with this biology, liver metastasis was more frequent in the *TP53* mutation group than in the WT group (58.3% vs 45.2%, *P* < 0.01), supporting the role of *TP53* mutations in promoting metastatic spread.

Genomic co-alteration patterns differed substantially between the *TP53* mutation group and the WT group. ERBB2, CDKN2A, SMAD4, PTEN, and CCNE1 alterations were enriched in the *TP53* mutation group, whereas IDH1 and *BAP1* mutations and *FGFR2* fusions were more frequent in the WT group. These findings are consistent with previous reports showing mutual exclusivity between *TP53* mutations *and IDH1/BAP1* mutations and *FGFR2* fusions in BTC [22, 23], reflecting distinct molecular subtypes. Leveraging a large, multi-institutional real-world dataset, we validate these genetic patterns and underscore the need for molecular stratification. We also characterized the spectrum of *TP53* variants. Missense mutations predominated, with hotspots R175, Y220, R273, and R282, consistent with profiles in other solid tumors. These hotspot variants are known to exert not only loss-of-function but also gain-of-function activities, including enhanced invasion and migration, EMT, metabolic reprogramming, and resistance to therapy [24]. Notably, Y220C creates a druggable pocket and is currently the focus of targeted stabilizer development (NCT04585750), underscoring its potential clinical relevance. Given this biological heterogeneity, the functional subclassification of *TP53* alterations may also be relevant to clinical behavior and treatment response in BTC.

A key finding of this study was the significant interaction between *TP53* mutation status and treatment effect on PFS in the tissue-based cohort, observed both in the overall cohort and in the MSS subset. In the non-ICI group, the *TP53* mutation group showed worse outcomes compared with the WT group, whereas in the ICI cohort, the *TP53* mutation group showed a trend toward better responses than the WT group. These findings suggest that although the *TP53* mutation group is associated with aggressive tumor biology and poor prognosis under conventional systemic therapy, it may also represent a subgroup that derives greater relative benefit from ICI-containing treatment. Similar, albeit less consistent, directional trends were observed in the plasma ctDNA-based cohort, supporting the overall robustness of this signal across sample types. One possible explanation for this apparent paradox is that *TP53* mutations may simultaneously drive aggressive disease behavior and alter the tumor immune microenvironment in ways that enhance responsiveness to ICI. Consistent with this, the *TP53* mutation group exhibited significantly lower TIDE scores than WT group, suggesting reduced immune dysfunction and exclusion. Furthermore, a multi-omics study of ICC reported that *TP53* mutations were enriched in the immune-classical subtype with higher T-cell infiltration and an inflamed tumor microenvironment [25]. In NSCLC, *TP53* mutations similarly associate with high immune infiltration and increased tumor mutational burden, with prolonged PFS and OS on ICI [18]. Nevertheless, despite these findings suggesting that the *TP53* mutation group may be more responsive to ICI, our GSEA revealed enrichment of pathways such as oxidative phosphorylation, hypoxia, EMT, and MYC targets, which have been implicated in immune evasion and ICI resistance in other cancers [26–28]. This paradox likely reflects the biological heterogeneity of *TP53*-mutated tumors: while these tumors may be more responsive to ICI overall, concurrent activation of resistance mechanisms could limit the extent of benefit. From a therapeutic perspective, the *TP53* mutation group represent a potentially ICI-sensitive subgroup, but rational combination strategies—such as targeting metabolic or hypoxia-related pathways—may be required to maximize benefit.

This study has several limitations. First, this retrospective design may introduce inherent selection biases. However, we sought to mitigate this limitation by integrating large, multi-institutional datasets from Japan and the U.S. Second, the ICI-containing subgroup was relatively small, limiting the statistical power to detect differences. Nevertheless, the treatment–*TP53* interaction reached statistical significance in multivariable Cox analyses, supporting the robustness of this finding. Third, although functional subclassification of *TP53* alterations may be clinically relevant, the number of gain-of-function cases was limited in our cohort, precluding a reliable analysis according to functional subtype. This issue should be addressed in future studies with larger sample sizes. Fourth, transcriptomic analyses relied on a small WTS (*n* = 59), so the findings should be interpreted cautiously. Notably, this subset did not include any MSI-H cases, which allowed us to assess the predictive significance of *TP53* mutations with greater clarity, independent of this well-established determinant of ICI efficacy. Fifth, regional differences in treatment standards and access to ICI between Japan and the U.S. may have influenced outcomes. Yet, the inclusion of both regions strengthens the generalizability. Finally, the subgroup analysis including both non-ICI and ICI cohorts should be interpreted cautiously, as these groups likely differed in treatment era and patient selection. In particular, the non-ICI cohort may have included patients treated before the widespread use of ICIs, as well as patients receiving GCS for conversion purposes, who may have had better performance status or lower tumor burden, thereby introducing additional selection bias. Moreover, 31 of 219 patients (14.2%) in the non-ICI cohort subsequently received ICI-containing therapy in later lines, which may have confounded the interpretation of overall survival.

## Conclusion

*TP53* mutations in advanced BTC were associated with poor prognosis under systemic therapy and may predict enhanced benefit from ICI. In an era where ICI is becoming standard of care, these findings underscore the need for biomarker-driven patient stratification to optimize treatment strategies in BTC.

## Supplementary Information

Below is the link to the electronic supplementary material.

Fig. S1 Kaplan–Meier curves for OS (A) and PFS (B) according to the ctDNA-based *TP53* mutation status in patients treated with first-line systemic therapy, and multivariable analyses conducted using Cox proportional hazards models to predict factors determining the overall survival (C) and progression-free survival (D) in patients with advanced biliary tract cancer. OS, overall survival; PFS, progression-free survival; ECC, extrahepatic cholangiocarcinoma; MSI-H, microsatellite instability- high; MSS, microsatellite- stable; WT, wild-type (DOCX 19 KB)

Fig. S2 ORR and DCR stratified by the tissue-based *TP53* status in patients treated with non-ICI and ICI-containing regimens. (A) ORR and DCR in the non-ICI cohort (*n* = 219), showing no significant differences between the WT and the *TP53* mutation groups. (B) ORR and DCR in the ICI cohort (*n* = 92), where the *TP53* mutation group showed a trend toward higher response rates, although the difference was not statistically significant (*P* = 0.35), and a significantly higher DCR (*P* = 0.03). ORR, objective response rate; DCR, disease control rate; WT, wild-type; ICI, immune checkpoint inhibitor (PPTX 419 KB)

Fig. S3 ORR and DCR stratified by plasma ctDNA-based *TP53* status in patients treated with non-ICI and ICI-containing regimens. (A) ORR and DCR in the non-ICI cohort, showing no significant differences between the WT and the *TP53* mutation groups. (B) ORR and DCR in the ICI cohort, where the *TP53* mutation group showed numerically higher ORR and DCR than the WT group, although neither difference reached statistical significance (ORR, *P* = 0.08; DCR, *P* = 0.70). ORR, objective response rate; DCR, disease control rate; WT, wild-type; ICI, immune checkpoint inhibitor (PPTX 167 KB)

Fig. S4 Kaplan–Meier survival curves for OS and PFS according to plasma ctDNA-based *TP53* mutation status in patients with advanced biliary tract cancer stratified by first-line treatment. In the non-ICI cohort, patients in the *TP53* mutation group showed numerically shorter OS (median, 15.9 vs. 20.5 months; *P* = 0.059; A) and significantly shorter PFS (median, 5.6 vs. 6.9 months; *P* = 0.025; C) than those in the WT group. In the ICI-containing regimen cohort, no significant difference in OS was observed (median, 25.3 months vs. NR; *P* = 0.614; B), and although PFS was numerically longer in the *TP53* mutation group, the difference was not statistically significant (median, 6.4 vs. 5.6 months; *P* = 0.158; D). OS, overall survival; PFS, progression-free survival; WT, wild-type; HR, hazard ratio; CI, confidence interval; NR, not reached (PPTX 167 KB)

Fig. S5 Forest plot of the treatment effect on the overall survival (A) and progression-free survival (B) in subgroup analyses in the plasma ctDNA-based cohort. HR, hazard ratio; CI, confidence interval; MSI-H, microsatellite instability-high; MSS, microsatellite-stable; ICI, immune checkpoint inhibitor (PPTX 310 KB)

Fig. S6 Transcriptomic correlates of the tissue-based *TP53* status. (A) The waterfall plot shows higher TIDE scores in the WT group and lower scores in the *TP53* mutation group. (B) The violin plot confirms this difference, with the *TP53* mutation group showing significantly lower TIDE scores (*P* = 0.01). (C) GSEA showing hallmark pathways enriched in the *TP53* mutation group, including oxidative phosphorylation, epithelial–mesenchymal transition, and hypoxia. TIDE, tumor immune dysfunction and exclusion; WT, wild-type; FWER, Family-Wise Error Rate; NES, normalized enrichment scores; GSEA, gene set enrichment analysis (PPTX 557 KB)

Supplementary file7 (PPTX 327 KB)

Supplementary file8 (DOCX 25 KB)

Supplementary file9 (DOCX 22 KB)

Supplementary file10 (DOCX 22 KB)

## Data Availability

A data sharing statement accompanies this article. For SCRUM-Japan GOZILA and MONSTAR-SCREEN, primary analyses are ongoing; upon completion, genomic data will be deposited in an appropriate public repository. Individual-level clinical data are not publicly available. Deidentified clinical data may be shared on reasonable request to the corresponding author, subject to steering-committee approval and institutional/ethical policies, including potential IP considerations.

## References

[1] Valle JW, Kelley RK, Nervi B, et al. Biliary tract cancer. Lancet. 2021;397:428–44. 10.1016/S0140-6736(21)00153-7 33516341

[2] Banales JM, Cardinale V, Carpino G, et al. Cholangiocarcinoma: current knowledge and future perspectives consensus statement from the European Network for the Study of Cholangiocarcinoma (ENS-CCA). Nat Rev Gastroenterol Hepatol. 2016;13:261–80. 10.1038/nrgastro.2016.51 27095655

[3] Zhang X-F, Beal EW, Bagante F, et al. Early versus late recurrence of intrahepatic cholangiocarcinoma after resection with curative intent. Br J Surg. 2018;105:848–56. 10.1002/bjs.10676 29193010

[4] Kelley RK, Ueno M, Yoo C, et al. Pembrolizumab in combination with gemcitabine and cisplatin compared with gemcitabine and cisplatin alone for patients with advanced biliary tract cancer (KEYNOTE-966): a randomised, double-blind, placebo-controlled, phase 3 trial. Lancet. 2023;401:1853–65. 10.1016/S0140-6736(23)00727-4 37075781

[5] Oh D-Y, He AR, Qin S, et al. A phase 3 randomized, double-blind, placebo-controlled study of durvalumab in combination with gemcitabine plus cisplatin (GemCis) in patients (pts) with advanced biliary tract cancer (BTC): TOPAZ-1. J Clin Oncol. 2022;40:378–378.

[6] Piha-Paul SA, Oh D-Y, Ueno M, et al. Efficacy and safety of pembrolizumab for the treatment of advanced biliary cancer: results from the KEYNOTE-158 and KEYNOTE-028 studies. Int J Cancer. 2020;147:2190–8. 10.1002/ijc.33013 32359091

[7] Lamarca A, Barriuso J, McNamara MG, et al. Biliary tract cancer: state of the art and potential role of DNA damage repair. Cancer Treat Rev. 2018;70:168–77. 10.1016/j.ctrv.2018.09.002 30218788

[8] Le DT, Durham JN, Smith KN, et al. Mismatch repair deficiency predicts response of solid tumors to PD-1 blockade. Science. 2017;357:409–13. 10.1126/science.aan6733 PMC5576142 28596308

[9] Marabelle A, Le DT, Ascierto PA, et al. Efficacy of Pembrolizumab in patients with noncolorectal high microsatellite instability/mismatch repair-deficient cancer: results from the phase II KEYNOTE-158 study. J Clin Oncol. 2020;38:1–10. 10.1200/JCO.19.02105 PMC8184060 31682550

[10] Goeppert B, Roessler S, Renner M, et al. Mismatch repair deficiency is a rare but putative therapeutically relevant finding in non-liver fluke associated cholangiocarcinoma. Br J Cancer. 2019;120:109–14. 10.1038/s41416-018-0199-2 PMC6325153 30377340

[11] Assoun S, Theou-Anton N, Nguenang M, et al. Association of TP53 mutations with response and longer survival under immune checkpoint inhibitors in advanced non-small-cell lung cancer. Lung Cancer. 2019;132:65–71. 10.1016/j.lungcan.2019.04.005 31097096

[12] Jiang P, Gu S, Pan D, et al. Signatures of T cell dysfunction and exclusion predict cancer immunotherapy response. Nat Med. 2018;24:1550–8. 10.1038/s41591-018-0136-1 PMC6487502 30127393

[13] Liberzon A, Birger C, Thorvaldsdóttir H, et al. The molecular signatures database (MSigDB) hallmark gene set collection. Cell Syst. 2015;1:417–25. 10.1016/j.cels.2015.12.004 PMC4707969 26771021

[14] Eisenhauer EA, Therasse P, Bogaerts J, et al. New response evaluation criteria in solid tumours: revised RECIST guideline (version 1.1). Eur J Cancer. 2009;45:228–47. 10.1016/j.ejca.2008.10.026 19097774

[15] Lin J, Cao Y, Yang X, et al. Mutational spectrum and precision oncology for biliary tract carcinoma. Theranostics. 2021;11:4585–98. 10.7150/thno.56539 PMC7978308 33754015

[16] Chae H, Kim D, Yoo C, et al. Therapeutic relevance of targeted sequencing in management of patients with advanced biliary tract cancer: DNA damage repair gene mutations as a predictive biomarker. Eur J Cancer. 2019;120:31–9. 10.1016/j.ejca.2019.07.022 31476489

[17] Lofthouse M. TP53 mutations as a prognostic factor in women with breast cancer. Nat Clin Pract Oncol. 2005;2:428–9.

[18] Mathiot L, Nigen B, Goronflot T, et al. Prognostic impact of TP53 mutations in metastatic nonsquamous non-small-cell lung cancer. Clin Lung Cancer. 2024;25:244-253.e2. 10.1016/j.cllc.2023.12.004 38218680

[19] Javle M, Bekaii-Saab T, Jain A, et al. Biliary cancer: utility of next-generation sequencing for clinical management. Cancer. 2016;122:3838–47. 10.1002/cncr.30254 27622582

[20] Olivier M, Hollstein M, Hainaut P. TP53 mutations in human cancers: origins, consequences, and clinical use. Cold Spring Harb Perspect Biol. 2010;2:a001008. 10.1101/cshperspect.a001008 PMC2827900 20182602

[21] Wang D, Nakayama M, Hong CP, et al. Gain-of-function p53 mutation acts as a genetic switch for TGFβ signaling-induced epithelial-to-mesenchymal transition in intestinal tumors. Cancer Res. 2024;84:56–68. 10.1158/0008-5472.CAN-23-1490 PMC10758690 37851521

[22] Jusakul A, Cutcutache I, Yong CH, et al. Whole-genome and epigenomic landscapes of etiologically distinct subtypes of cholangiocarcinoma. Cancer Discov. 2017;7:1116–35. 10.1158/2159-8290.CD-17-0368 PMC5628134 28667006

[23] Nakamura H, Arai Y, Totoki Y, et al. Genomic spectra of biliary tract cancer. Nat Genet. 2015;47:1003–10. 10.1038/ng.3375 26258846

[24] Baugh EH, Ke H, Levine AJ, et al. Why are there hotspot mutations in the TP53 gene in human cancers? Cell Death Differ. 2018;25:154–60. 10.1038/cdd.2017.180 PMC5729536 29099487

[25] Martin-Serrano MA, Kepecs B, Torres-Martin M, et al. Novel microenvironment-based classification of intrahepatic cholangiocarcinoma with therapeutic implications. Gut. 2023;72:736–48. 10.1136/gutjnl-2021-326514 PMC10388405 35584893

[26] Tian J, Luo J, Zeng X, et al. Targeting oxidative phosphorylation to increase the efficacy of immune-combination therapy in renal cell carcinoma. J Immunother Cancer. 2024. 10.1136/jitc-2023-008226. PMC10868282 38355278

[27] Robles-Oteíza C, Hastings K, Choi J, et al. Hypoxia is linked to acquired resistance to immune checkpoint inhibitors in lung cancer. J Exp Med. 2025. 10.1084/jem.20231106. PMC11602551 39585348

[28] Jeong H, Koh J, Kim S, et al. Epithelial−mesenchymal transition induced by tumor cell-intrinsic PD-L1 signaling predicts a poor response to immune checkpoint inhibitors in PD-L1-high lung cancer. Br J Cancer. 2024;131:23–36. 10.1038/s41416-024-02698-4 PMC11231337 38729997

