# Supplementary material for: Clinico-molecular characteristics and enhanced efficacy of immune checkpoint inhibitors in TP53-mutated advanced biliary tract cancer
Source: J Gastroenterol. 2026 May 25;61(11):1632–42. doi: 10.1007/s00535-026-02439-9 (PMC13695642; doi:10.1007/s00535-026-02439-9)
Supplement: Supplementary file 1 [file 535_2026_2439_MOESM10_ESM.docx]

|  | Non-ICI cohort  (n=454) | ICI cohort  (n=31) | | *P-value* | |  |  |
| --- | --- | --- | --- | --- | --- | --- | --- |
| Age (years), *n* (%) |  | |  | |  | |  |
| Median [range] | 68 [31, 84] | | 65 [50, 81] | | 0.85 | |  |
| <70 | 265 (58.4) | | 17 (54.8) | | 0.84 | |  |
| ≥70 | 189 (41.6) | | 14 (45.2) | |  | |  |
| Sex, *n* (%) |  | |  | |  | |  |
| Male | 265 (58.4) | | 12 (38.7) | | 0.04 | |  |
| Female | 189 (41.6) | | 19 (61.3) | |  | |  |
| Primary tumor site, *n* (%) |  | |  | |  | |  |
| Extrahepatic bile duct | 104 (22.9) | | 7 (22.6) | | 0.14 | |  |
| Gallbladder | 140 (30.8) | | 10 (32.3) | |  | |  |
| Intrahepatic bile duct | 156 (34.4) | | 14 (45.2) | |  | |  |
| Ampulla of Vater | 54 (11.9) | | 0 (0) | |  | |  |
| Tumor status, *n* (%) |  | |  | |  | |  |
| Recurrence | 170 (37.5) | | 1 (3.2) | | <0.01 | |  |
| Locally advanced | 28 (6.2) | | 4 (12.9) | |  | |  |
| Metastatic | 255 (56.3) | | 26 (83.9) | |  | |  |
| MSI-High detected, n (%) | 19 (5.1) | | 2 (6.5) | | 0.67 | |  |
| Site of metastasis, n (%) |  | |  | |  | |  |
| liver | 236 (52.0) | | 14 (45.2) | | 0.58 | |  |
| lymph node | 207 (45.6) | | 13 (41.9) | | 0.71 | |  |
| lung | 108 (23.8) | | 4 (12.9) | | 0.19 | |  |
| peritoneum | 76 (16.7) | | 2 (6.5) | | 0.20 | |  |
| Others | 43 (9.5) | | 3 (9.7) | | 1 | |  |
| *TP53* mutation (Yes), *n* (%) | 232 (51.1) | | 15 (48.4) | | 0.85 | |  |
| ICI, immune checkpoint inhibitor; WT, wild type; MSI, microsatellite status instability; NGS, next-generation sequencing | | | | | | | |
