# Supplementary material for: Clinico-molecular characteristics and enhanced efficacy of immune checkpoint inhibitors in TP53-mutated advanced biliary tract cancer
Source: J Gastroenterol. 2026 May 25;61(11):1632–42. doi: 10.1007/s00535-026-02439-9 (PMC13695642; doi:10.1007/s00535-026-02439-9)
Supplement: Supplementary file 2 [file 535_2026_2439_MOESM1_ESM.docx]

|  | WT  (n=104) | *TP53-*mutated  (n=111) | | *P-value* | |  |  |
| --- | --- | --- | --- | --- | --- | --- | --- |
| ICI cohort, *n* (%) |  | |  | |  | |  |
| Subsequent systemic therapy | 11 (64.7%) | | 16 (80.0%) | | 0.460 | |  |
| BSC | 6 (35.3%) | | 4 (20.0%) | |  | |  |
| Non-ICI cohort, *n* (%) |  | |  | |  | |  |
| Subsequent systemic therapy | 47 (56.0%) | | 59 (64.8%) | | 0.279 | |  |
| BSC | 37 (44.0%) | | 32 (35.2%) | |  | |  |
| WT, wild type; ICI, immune checkpoint inhibitor; BSC, best supportive care | | | | | | | |
