# Supplementary material for: Clinico-molecular characteristics and enhanced efficacy of immune checkpoint inhibitors in TP53-mutated advanced biliary tract cancer
Source: J Gastroenterol. 2026 May 25;61(11):1632–42. doi: 10.1007/s00535-026-02439-9 (PMC13695642; doi:10.1007/s00535-026-02439-9)
Supplement: Supplementary file 3 [file 535_2026_2439_MOESM8_ESM.docx]

|  | WT  (n=252) | *TP53-*mutated  (n=262) | | *P-value* | |  |  |
| --- | --- | --- | --- | --- | --- | --- | --- |
| Age (years), *n* (%) |  | |  | |  | |  |
| Median [range] | 67 [35, 82] | | 69 [31, 84] | | 0.01 | |  |
| <70 | 159 (63.1) | | 142 (54.2) | | 0.05 | |  |
| ≥70 | 93 (36.9) | | 120 (45.8) | |  | |  |
| Sex, *n* (%) |  | |  | |  | |  |
| Male | 153 (60.7) | | 142 (54.2) | | 0.15 | |  |
| Female | 99 (39.3) | | 120 (45.8) | |  | |  |
| Primary tumor site, *n* (%) |  | |  | |  | |  |
| Extrahepatic bile duct | 55 (21.8) | | 62 (23.7) | | <0.01 | |  |
| Gallbladder | 61 (24.2) | | 96 (36.6) | |  | |  |
| Intrahepatic bile duct | 115 (45.6) | | 70 (26.7) | |  | |  |
| Ampulla of Vater | 21 (8.3) | | 34 (13.0) | |  | |  |
| Tumor status, *n* (%) |  | |  | |  | |  |
| Recurrence | 85 (33.9) | | 93 (35.5) | | 0.61 | |  |
| Locally advanced | 21 (8.4) | | 16 (6.1) | |  | |  |
| Metastatic | 145 (57.8) | | 153 (58.4) | |  | |  |
| MSI-High detected, n (%) | 7 (3.3) | | 14 (6.4) | | 0.179 | |  |
| Site of metastasis, n (%) |  | |  | |  | |  |
| liver | 112 (44.4) | | 156 (59.5) | | <0.01 | |  |
| lymph node | 116 (46.0) | | 116 (44.3) | | 0.72 | |  |
| lung | 63 (25.0) | | 52 (19.8) | | 0.17 | |  |
| peritoneum | 41 (16.3) | | 39 (14.9) | | 0.72 | |  |
| Others | 24 (9.5) | | 23 (8.8) | | 0.88 | |  |
| Site, n (%) |  | |  | |  | |  |
| Japan | 249 (98.8) | | 254 (96.9) | | 0.22 | |  |
| US | 3 (1.2) | | 8 (3.1) | |  | |  |
| 1^st^ line regimen |  | |  | |  | |  |
| Triplet | 62 (24.6) | | 70 (26.7) | | 0.88 | |  |
| Gem+Cis+S-1 | 47 (18.7) | | 55 (21.0) | |  | |  |
| Gem+Cis+ICI | 15 (6.0) | | 15 (5.7) | |  | |  |
| Doublet | 161 (63.9) | | 161 (61.5) | |  | |  |
| Gem+Cis | 150 (59.5) | | 151 (57.6) | |  | |  |
| Gem+S-1 | 11 (4.4) | | 10 (3.8) | |  | |  |
| Singlet | 15 (6.0) | | 16 (6.1) | |  | |  |
| S-1 | 7 (2.8) | | 0 (0) | |  | |  |
| Gem | 2 (1.3) | | 11 (4.2) | |  | |  |
| ICI | 1 (0.4) | | 0 (0) | |  | |  |
| Unknown | 13 (5.2) | | 12 (4.6) | |  | |  |
| WT, wild type; MSI, microsatellite status instability; NGS, next-generation sequencing; Gem, gemcitabine; Cis, cisplatin; ICI, immune checkpoint inhibitor | | | | | | | |
