# Supplementary material for: Clinico-molecular characteristics and enhanced efficacy of immune checkpoint inhibitors in TP53-mutated advanced biliary tract cancer
Source: J Gastroenterol. 2026 May 25;61(11):1632–42. doi: 10.1007/s00535-026-02439-9 (PMC13695642; doi:10.1007/s00535-026-02439-9)
Supplement: Supplementary file 4 [file 535_2026_2439_MOESM9_ESM.docx]

|  | Non-ICI cohort  (n=219) | ICI cohort  (n=92) | | *P-value* | |  |  |
| --- | --- | --- | --- | --- | --- | --- | --- |
| Age (years), *n* (%) |  | |  | |  | |  |
| Median [range] | 68 [26, 84] | | 68 [33, 82] | | 0.97 | |  |
| <70 | 129 (58.9) | | 50 (54.9) | | 0.61 | |  |
| ≥70 | 90 (41.1) | | 41 (45.1) | |  | |  |
| Sex, *n* (%) |  | |  | |  | |  |
| Male | 124 (56.6) | | 38 (41.3) | | 0.02 | |  |
| Female | 95 (43.4) | | 54 (58.7) | |  | |  |
| Primary tumor site, *n* (%) |  | |  | |  | |  |
| Extrahepatic bile duct | 51 (23.3) | | 20 (21.7) | | 0.04 | |  |
| Gallbladder | 62 (28.3) | | 22 (23.9) | |  | |  |
| Intrahepatic bile duct | 85 (38.8) | | 48 (52.2) | |  | |  |
| Ampulla of Vater | 21 (9.6) | | 2 (2.2) | |  | |  |
| Tumor status, *n* (%) |  | |  | |  | |  |
| Recurrence | 81 (37.0) | | 24 (26.1) | | 0.13 | |  |
| Locally advanced | 17 (7.8) | | 11 (12.0) | |  | |  |
| Metastatic | 121 (55.3) | | 57 (62.0) | |  | |  |
| MSI-High detected, n (%) | 4 (2.0) | | 1 (1.1) | | 1.00 | |  |
| Site of metastasis, n (%) |  | |  | |  | |  |
| liver | 102 (46.6) | | 39 (42.4) | | 0.53 | |  |
| lymph node | 82 (37.4) | | 28 (30.4) | | 0.25 | |  |
| lung | 30 (13.7) | | 17 (18.5) | | 0.30 | |  |
| peritoneum | 25 (11.4) | | 14 (15.2) | | 0.35 | |  |
| Others | 8 (3.7) | | 7 (7.6) | | 0.15 | |  |
| *TP53* mutation (Yes), *n* (%) | 117 (53.4) | | 50 (54.3) | | 0.90 | |  |
| ICI, immune checkpoint inhibitor; WT, wild type; MSI, microsatellite status instability; NGS, next-generation sequencing | | | | | | | |
